# Regulation of podocalyxin expression in the kidney of streptozotocin-induced diabetic rats with Chinese herbs (Yishen capsule)

**DOI:** 10.1186/1472-6882-13-76

**Published:** 2013-04-05

**Authors:** Jingai Fang, Hongkun Wei, Yanyan Sun, Xiaodong Zhang, Wenyuan Liu, Qintao Chang, Ruihua Wang, Yuewen Gong

**Affiliations:** 1 Department of Nephrology, The First Affiliated Hospital to the Shanxi Medical University, 56 Xinjian South Road, Taiyuan, Shanxi Province, 030001, People’s Republic of China; 2 Faculty of Pharmacy, University of Manitoba, Winnipeg, R3E 0 T5, Canada

## Abstract

**Background:**

Diabetic nephropathy is an emergent issue in China with increase in patients with type II diabetes. There are several successful Chinese herbal products for the treatment of patients with diabetic nephropathy in China. However, the mechanisms mediating the biological activity of these products are still unclear. Podocalyxin is a sialoprotein critical to maintaining integrity of filtration function of glomerulus.

**Methods:**

By employing streptozotocin-induced diabetic rats and a Chinese herb formulation (Yishen capsule), we examined the regulation of podocalyxin expression in the kidney by Yishen capsule through immunofluorescent staining and reverse transcriptase polymerase chain reaction.

**Results:**

After injection of STZ, there were significant increase in both blood glucose and urinary protein. Serum creatinine and BUN were also increased in rats with injection of STZ. Moreover, expression of podocalyxin in the glomerulus was gradually reduced after injection of STZ. There was also a loss of podocyte foot processes in the glomerular basement membrane. However, Yishen capsule or benazepril was able to restore the expression of podocalyxin and podocyte foot processes in the kidney. Although Yishen capsule could reduce urinary protein level, it has little effect on blood glucose level in the rats injected with STZ.

**Conclusions:**

Yishen capsule could attenuate the loss of podocalyxin in the glomerulus of rats injected with STZ.

## Background

Recent investigation revealed that glomerular epithelial cells or podocytes are the main cell type involved in the development of diabetic nephropathy, which is one of the most important microvascular complications associated with type 2 diabetic patients [1]. Diabetic nephropathy refers to a spectrum of renal diseases in diabetic patients from microalbuminuria to macroalbuminuria and progressive decline in glomerular filtration rate [2]. Moreover, diabetic nephropathy is the most common cause of the end-stage of renal disease (ESRD), which is about 40% of patients receiving renal replacement therapy in Asia [3]. Pathological changes of kidney in diabetic nephropathy include the thickness of glomerular basement membranes and progressive accumulation of extracellular matrix components, especially the change of podocytes’ ultra-structure and function.

Podocytes are specialized cells located in the glomerular basement. Interaction between podocyte foot processes controls the glomerular filtration. There are several proteins on podocyte membrane that are important in regulation of the glomerular filtration such as nephrin, podocin and podocalyxin [4,5]. Podocalyxin is an integral membrane protein on the foot processes of kidney podocytes [6]. It is a sialoprotein contributing to the negative charge of glomerular membrane. Knockout podocalyxin in mice resulted in early death after birth due to the failure of podocytes to develop foot processes [7]. Regulation of podocalyxin in the developing kidney is mediated by transcription factors - Wilms’ tumor suppressor protein (WT1) and Sp1 [8,9]. However, regulation of podocalyxin in adult kidney remains to be investigated.

Traditional Chinese medicine has been widely used in China for the treatment of diabetes and diabetic complications. Several studies reported that Chinese herbal medicines were used to improve conditions of patients with diabetic nephropathy [10] and it becomes more promising in the treatment of diabetic nephropathy [11]. From our clinical experience, we also developed a formulation for patients with diabetic nephropathy, which is named as Yishen capsule. Yishen capsule consists of five Chinese herbs (Astragalus menbranaceus, Angelica sinensis, Euryale ferox, Alisma orientale, and Rhodiola rosea). Astragalus membranaceus and angelica sinensis have been shown to regulate immune and oxidative system [12]. In addition astragalus membranaceus has been shown to improve patients’ proteinuria with idiopathic membranous nephropathy [13,14]. Euryale ferox and alisma orientale also have antioxidative activity [15,16]. Salidroside from rhodiola rosea has anti-inflammatory activity by inhibiting production of tumor necrosis factor alpha and interleukin-6 [17]. Clinical observation of this formulation revealed that it could reduce proteinuria, protect renal function, and delay progression of early diabetic nephropathy [18]. However, the molecular and cellular mechanisms of Yishen capsule for the treatment of diabetic nephropathy still remain unclear. Therefore, in the current study, we employs diabetic nephropathy model to investigate the effects of Yishen capsule on proteinuria, glomerular filtration and podocalyxin expression in the kidney.

## Methods

Materials: streptozotocin (STZ) and other chemical reagents were purchased from Sigma Co. (St. Louis, MO. USA). Rabbit anti-rat podocalyxin polyclonal antibody and FITC conjugated goat anti-rabbit IgG polyclonal antibody were purchased from Santa Cruz Biotechnology (Santa Cruz, CA USA). Hoechst-33258 and Benazepril hydrochloride were purchased from Biyuntian (Biyuntian Biotechnology Co, Shanghai, CN) and Novartis pharmaceutical (Novartis pharmaceutical Inc., Beijing, China) respectively.

Components of Yishen capsule: Yishen capsule was prepared by the Department of Pharmacy of the First Affiliated Hospital of the Shanxi Medical University. It consists of Astragalus membranaceus, Angelica sinensis, Euryale ferox, Alisma orientale, Rhodiola rosea in ratio of 3:2:3:2:1. These herbs were boiled in water for 1 hour three times. The decoctions were combined and filtered through 3 M membranes. The decoction was then concentrated through vacuum evaporation to density of 1.20-1.24 at 70°C. The concentrated decoction was further spray-dried to particle, which was then used to fill capsule.

Streptozotocin (STZ) induced model of diabetic nephropathy: 48 Wistar rats weighing from 230-250 gram were purchased from the Experimental Animal Centre of the Shanxi Medical University. After one week, rats were randomly divided into the following groups: 1) normal control group (8 rats), 2) STZ diabetic group treated with saline (24 rats), 3) STZ diabetic group treated with Yishen capsule (8 rats) and 4) diabetic group treated with benazepril (8 rats). Rats in normal control group were treated with sham operation and injection of 10 mM sodium citrate buffer pH 4.5 once intraperitoneally one week after sham operation. Diabetic groups were treated with right uninephrectomy follow by single intraperitoneal injection of 35 mg/kg STZ in 10 mM sodium citrate buffer pH 4.5 one week after uninephrectomy. After one week of STZ injection, blood glucose level was examined with tail blood. When the blood glucose level reached 16.7 mmol/L, rats were confirmed to be at diabetic state. For group 2, rats were sacrificed at 0, 4 and 12 weeks after STZ injection with 8 rats in each time point. Normal saline, Yishen capsule (625 mg/kg/day) and benazepril (3.125 mg/kg/day) were gavaged to rats in each group once every day for 12 weeks respectively except for group 2 where some rats were sacrificed at 0 and 4 weeks after STZ. The dose used in the experiment was converted according to clinical dose in patients which is 100 mg/kg/day for Yishen capsule and 0.5 mg/kg/day for benazepril. In addition, previous studies also indicated that 625 mg/kg/day of Yishen capsule were efficient to regulate podocytes [19]. This study was approved by the Ethics Committee of the Shanxi Medical University in accordance with the Guideline for the Care and Use of Laboratory Animals.

Proteinuria: Twenty-four hours urinary samples were collected from rats in metabolic cages without restriction of water intake. Urines were collected at the end of 2, 4, 6, 8 and 12 weeks after STZ injection. After collection, urine volume was recorded and 4 ml of urine were centrifuged at 2,000 rpm for 10 minute. The supernatant were collected and kept at -20°C until protein determination, which was performed by the Bradford method [20].

Serum urine nitrogen (BUN) and creatinine (Cre) determination: At the end of experiment, blood samples (3 ml) were removed from the heart and rats were sacrificed. Blood were left at room temperature for 1 hour and then at 4°C for 6 hours. Blood samples were then centrifuged at 2,500 rpm for 15 minutes at 4°C. The supernatant was then stored at -20°C until BUN and Cre were determined by an automatic clinical chemistry analyzer (COBAS INTEGRA® 800 Roche).

Kidney histological examination: The kidneys were collected at the end of experiment. Part of the kidney was fixed in 10% neutral buffered formalin and embedded in paraffin. Sections about 2-3 μm thick were cut and mounted on glass slides and paraffin was depleted by xylene. The sections were then subjected to standard Periodic acid-Schiff (PAS) staining and were observed under light microscopy with investigators blinded to group identity.

Electron microscope analysis of the kidney podocytes foot processes: Part of the kidney was fixed in 2.5% glutaraldehyde for 2 hours and washed with PBS three times. Tissues were fixed in 10% osmium tetroxide for 2 hours and embedded in Epon812. After ultracut, section was stained with lead citrate and ultrastructure of the kidney was observed under Hitachi H-600 electron microscope (Hitachi Company, Japan) at magnification of 36,000X. Thirty visions were observed for each section to insure that one hundred vision fields were obtained for each rat.

Immunofluorescent examination of podocalyxin in the kidney: Part of the kidney was snap-frozen in n-hexane cooled to -70°C and then 4 μm-thick sections were cut with a cryostat (Leica, CM1850, German). After the sections were mounted on glass slide and washed three times with PBS, the sections were incubated with 3% BSA for 30 minutes to block non-specific binding. The primary antibody against podocalyxin was incubated with the kidney section overnight at 4°C. The PBS was used for negative control. After washing three times with PBS of 10 minutes each time, 50-100 μl of FITC conjugated goat anti-rabbit secondary antibody will be incubated with the section for 1 hour under the dark condition. The sections were then dried, sealed and stored at 4°C until observation under fluorescence microscopy (Optima 6.5, Media Cybernetics Inc. Silver Spring, MD, USA) with 100X objective. The densities of flurorescence were determined by computer analysis with Image-Pro Plus 5.0 (Media Cybernetics, USA).

Reverse transcriptase-polymerase chain reaction (RT-PCR): Total RNA from kidney was isolated by using TRIzol reagent (Invitrogen, USA) according to the manufacturer's protocol. Two μg RNA was employed for the first strand cDNA synthesis by using moloney murine leukemia virus reverse transcriptase. PCR amplification was performed using a thermocycler (Bio-Rad Laboratory, USA) by the following steps: denaturing at 94°C for 5 min followed by 35 cycles of denaturing at 94°C for 30 seconds, annealing for 30 seconds at temperature 52°C for podocalyxin and 51°C for GAPDH, and extending at 72°C for 45 seconds. PCR was ended after elongation at 72°C for 10 min. The sequences of primers for podocalyxin are sense strand = ACC GGT CCT TAA TTG GTT CC and antisense strand = CCT TTG GCA GTT AGG AGC TG. Product length is 201 base pair. Primers sequences for GAPDH are sense strand = TGA ACG GGA AGC TCA CTG G and antisense strand = TCC ACC ACC CTG TTG CTG TA. Product length is 307 base pair. Densities of the bands were quantified using the Quantity One software (Bio-rad, USA), and results were represented as ratio of podocalyxin to GAPDH.

Statistical analysis: The data were represented as mean ± standard deviation (SD). Differences between control and treatment groups were analyzed by the ANOVA and Fisher's PLSD test as Post hoc test using the SPSS10.0 statistical analysis software. P value of less than 0.05 was considered as statistical significance.

## Results

### Blood glucose and microalbuminuria in STZ induced diabetic rats

**Figure 1:**
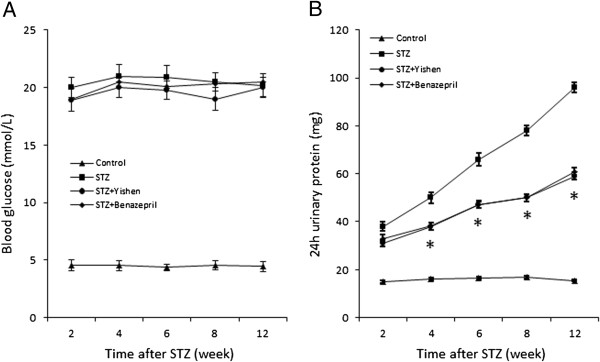
**Glucose and urinary albumin levels after STZ injection in rats.** After rats were injected with STZ, blood glucose level and urinary albumin level were determined at different time intervals as indicated. Blood glucose levels were significantly increased in rats with injection of STZ and treatments of Yishen or benazepril. Urinary protein was also gradually increased in rats with STZ injection. However, both Yishen and benazepril treatments were able to reduce the amount of protein in the urine but were not able to bring protein content back to its normal level. Data are expressed as mean ± SD from eight rats. * indicates p < 0.05 as compared to normal control group.

To examine early change of the kidney in diabetic nephropathy, we employed STZ induced diabetic model and examined the blood glucose and urinary albumin level at 2, 4, 6, 8 and 12 weeks after injection of STZ. As shown in Figure 1A, rats without injection of STZ had a normal blood glucose level around 5 mmol/L while rats with STZ injection had higher blood glucose level around 20 mmol/L. With treatment of Yishen capsule or benazepril, there was no statistically significant reduction of blood glucose level compared to that of STZ treatment alone. In addition, we measured 24 hours albumin in the urines of these rats and found that except normal control group, albumin content in the urines of all other groups was elevated two weeks after STZ injection (Figure 1B). Rats in STZ group showed gradual increased microalbuminuria from 2 weeks to 12 weeks after STZ injection. Although rats treated with Yishen capsule or benazepril also showed gradual increased microalbuminuria, the amount of albumin in the urines of these rats was significantly lower than that of rats in STZ group at 4, 6, 8, 10 and 12 weeks after STZ injection respectively.

### Expression of podocalyxin in STZ induced diabetic rats

**Figure 2:**
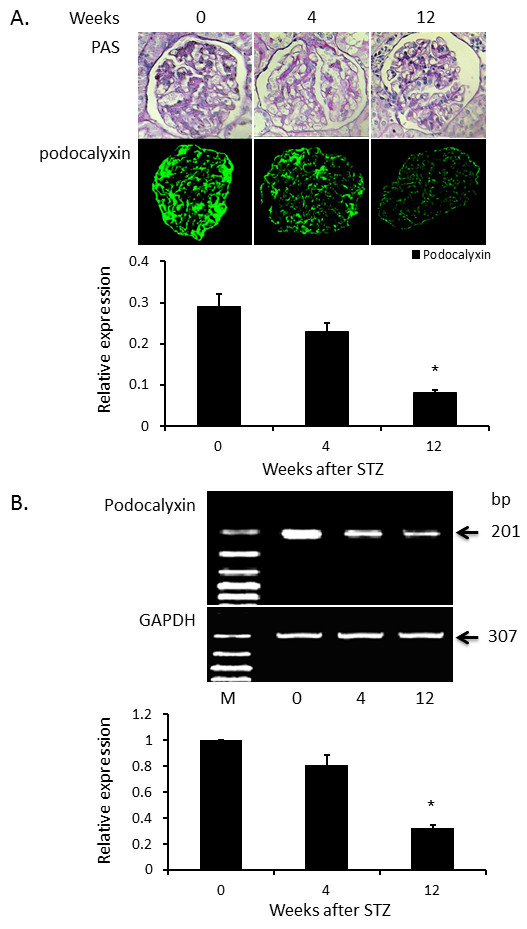
**Expression of podocalyxin in the kidney after STZ injection in rats.** Kidney histology and expression of podocalyxin were documented with HE staining, immunofluorescent staining of podocalyxin and RT-PCR analysis of podocalyxin mRNA respectively. As shown in panel **A**, glomerulus was damaged and podocalyxin fluorescent intensity was reduced (magnification x400). The expression of podocalyxin was gradually reduced from 0 to 12 weeks after STZ injection. The expression of podocalyxin was further documented by RT-PCR analysis of podocalyxin mRNA as displayed in panel **B**. Podocalyxin level at 12 weeks after STZ injection was about one third of normal value. Data are expressed as mean ± SD from eight rats. * indicates p < 0.05 as compared to normal control group.

We then sacrificed rats at 0, 4, and 12 weeks after STZ injection for examination of kidney histology and the expression of podocalyxin. As shown in Figure 2, glomerulus of the kidney at 12 weeks after STZ injection shows increased space within glomerulus. The expression of podocalyxin was documented by both immunofluorescent histology and RT-PCR analysis. As shown in Figure 2A, there was a gradual reduction of fluorescent intensity after STZ injection from 0 to 4 weeks to 12 weeks. The intensity of podocalyxin fluorescence at 12 weeks after STZ injection is only about one third of that before STZ injection. RT-PCR also documented a gradual reduction of podocalyxin mRNA level after STZ injection (Figure 2B). The amount of podocalyxin mRNA in the kidney at 12 weeks after STZ injection is also about one third of that before STZ injection.

### Modulation of podocalyxin by Yishen capsule in STZ induced diabetic rats

**Figure 3:**
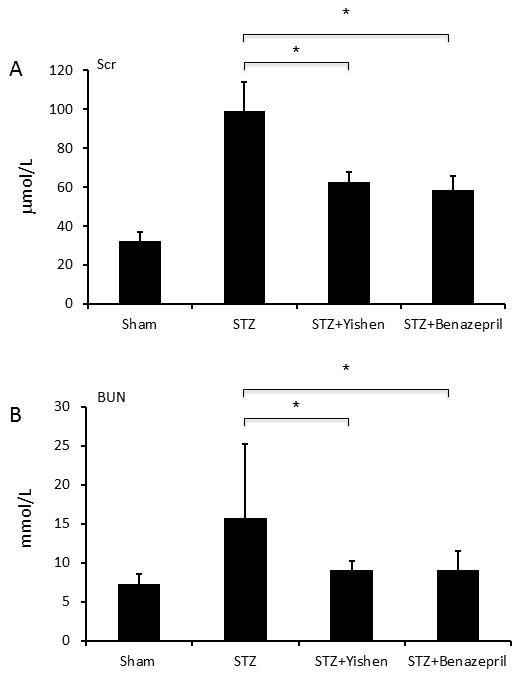
**Effect of Yishen capsule on serum creatinine and BUN levels in STZ induced diabetic rats.** Rat blood was prepared from different groups as indicated. Serum creatinine and BUN levels were determined as described in the materials and methods. Panel **A** displays the effect of Yishen capsule on serum creatinine in different groups. Panel **B** shows the effect of Yishen capsule on BUN levels in different groups. Data are expressed as mean ± SD from eight rats. * indicates p < 0.05 as compared to normal control group.

**Figure 4:**
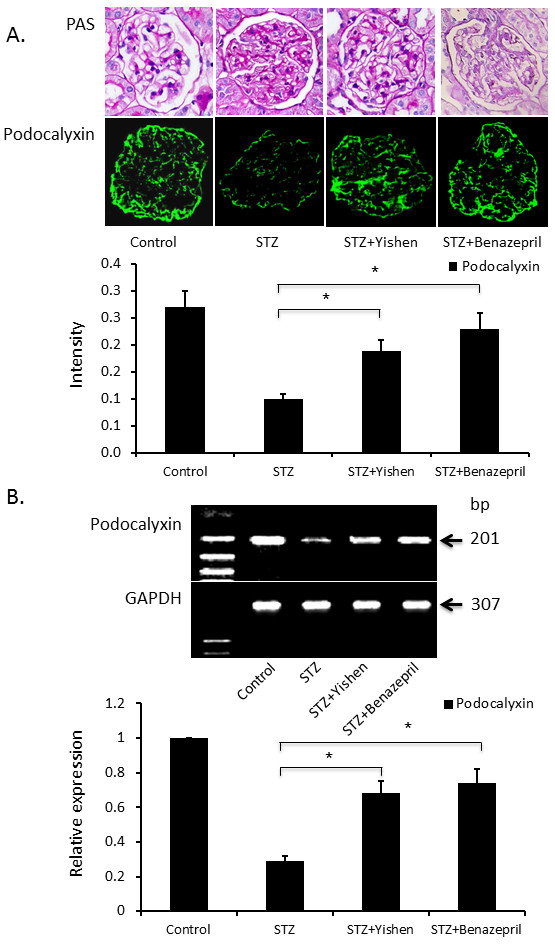
**Effect of Yishen capsule on the expression of podocalyxin in the kidney of STZ induced diabetic rats.** The regulation of podocalyxin in the glomerulus is displayed in panel **A** (podocalyxin fluorescence) and panel **B** (RT-PCR). Both Yishen capsule and benazepril were able to restore expression of podocalyxin in the glomerulus of rats with STZ injection. However, podocalyxin levels in Yishen capsule and/or benazepril treatment groups are still lower than that of normal group. Data are expressed as mean ± SD from eight rats. * indicates p < 0.05 as compared to normal control group.

**Figure 5:**
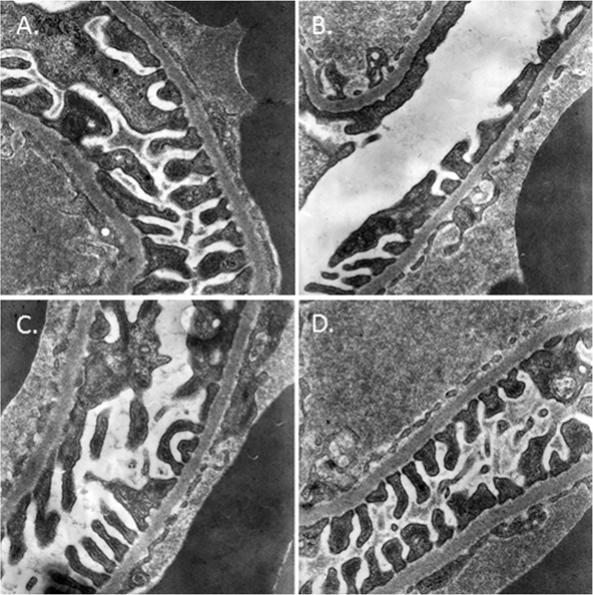
**Electron microscopy examination of the glomerular basement membrane in the kidney of rats.** Sections of kidney from different groups were analyzed with electron microscope (magnification x36,000). There was a significant reduction of podocyte foot processes in STZ injection groups. Panel **A** represents the kidney from normal rats. Panel **B** shows the kidney from STZ injected rats. Panel **C** and panel **D** display the kidney tissues from STZ injected rats with treatments of Yishen capsule or benazepril respectively.

After successful documentation of diabetes in rats and expression of podocalyxin in the kidney after STZ injection, we further examined the effects of Yishen capsule on STZ induced diabetes and expression of podocalyxin in the kidney. As shown in Figure 3, there was a significant increase in both Scr and BUN in the blood of rats in STZ group as compared to that of rats in normal group at 12 weeks after STZ injection. Moreover, treatment of Yishen capsule or benazepril significantly reduced both Scr and BUN in these rats. Regulation of podocalyxin expression by Yishen capsule or benazepril was documented at 12 weeks after STZ injection by immunofluorescent histology and RT-PCR analysis (Figure 4). Both Yishen capsule and benazepril significantly restored the expression of podocalyxin protein and mRNA in the kidney as documented by immunofluorescence (Figure 4A) and RT-PCR analyses (Figure 4B) respectively. Although the level of podocalyxin in Yishen treated rats was lower than that in benazepril treated rats, there was no statistically significant between these groups. Furthermore, we examined ultrastructure of the glomerular basement membrane. As shown in Figure 5, podocyte foot processes was inter-connected to each other in the glomerular basement membrane of normal kidney (Figure 5A). However, STZ treatment significantly reduced podocyte foot processes and there was a lot of space between basement membrane (Figure 5B). Moreover, treatment of either Yishen capsule or benazepril restored podocyte foot processes almost back to normal level (Figure 5C &5D).

## Discussion

Development of diabetic nephropathy is a complicated process, which involves in several factors such as glomerular mesangial cells and podocytes. Podocyte injury has been considered as one of the most important factors in diabetic nephropathy [21,22]. Several medicines that protect podocyte injury usually have a role in prevention of development of diabetic nephropathy. These medications are either conventional drugs or some herbs from traditional Chinese medicine [10,23]. In the current report, we employed Yishen capsule that have been documented to reduce progression of diabetic nephropathy in patients with type II diabetes [18], to investigate their mechanism of prevention of diabetic nephropathy. Yishen capsule includes an herb (*Astragalus membranaceus*), which is known to treat proteinuria in patients with diabetic nephropathy. However, molecular and cellular mechanism of these herbs to attenuate the development and progression of diabetic nephropathy are still unclear. Our study reveals that Chinese herbs could modulate the expression of podocalyxin in the kidney of streptozotocin-induced diabetic rats.

Podocalyxin is one of the major negatively charged glomerular proteins in the glomerular basement membrane. It located on the surface of podocytes that faces the urinary space. Podocalyxin also plays an important role in maintenance of the intricate glomerular podocyte for optimal filtration. Expression and function of podocalyxin changed in numerous human diseases and animal models of glomerular malfunction, such as diabetic nephropathy, puromycin aminonucleoside nephrosis and protamine sulfate perfusion. In these conditions, podocalyxin’s negative charge is neutralized, foot process architecture is disrupted, and slit diaphragms are displaced or completely replaced by leaky, discontinuous junctions [24]. Podocalyxin-knockout mice shew that podocalyxin maintains the unique podocyte morphology because in these mice, there were fewer major processes, and lack foot processes and slit diaphragms [7]. In our current study, we also observed similar morphological changes in the kidney of STZ-induced diabetic rats showing lack of foot processes and slit diaphragms. However, treatment with Yishen capsule corrected these defects. These modifications of the glomerulus were similar to those of benazepril. These changes could be due to regulation of podocalyxin expression by either Yishen capsule or benazepril as demonstrated in the current study.

The Yishen capsule is a formulation of traditional Chinese herbs created by Dr. Fang in 1995, which has been employed to treat patients with chronic kidney disease [25,26]. Among these herbs, astragalus and angelica have been demonstrated to protect heart and brain injury due to ischemia/reperfusion. Moreover, astragalus and angelica also promote tubular cell proliferation during renal ischemia/reperfusion injury. Although the mechanism involved in protection of ischemia/reperfusion injury by astragalus and angelica remain to be further investigation, the components of astragalus such as flavone, saponin and polysaccharide can inhibit lipid peroxidation and enhance anti-oxidative activity of cells [27,28]. Furthermore, astragalus induced complete remission of idiopathic membranous nephropathy [13,14]. In addition, angelica was able to reduce intracellular Ca2+ overload and one of its components – ferulic acid was also able to reduce production of superoxide in hepatocytes and prevent injury of lipid membrane after ischemia/reperfusion [29]. Other herbs in the formulation include euryale ferox, alisma orientale and rhodiola rosea. Both euryale ferox and alisma orientale have anti-oxidative activity [15,16] while a component of rhodiola rosea (salidroside) could inhibit inflammatory cytokines production such as tumor necrosis factor alpha and interleukin-6 [17]. Therefore, Yishen capsule has anti-oxidative and anti-inflammatory function.

Natural herb products as complementary therapies for chronic kidney disease have been widely investigated. Most of these therapies were focused on recovery of kidney function after ischemia/reperfusion injury of the kidney. For example, *Cordyceps sinesis* (dongchongxiacao), which is a blade-shaped fungus derived from larvae of *Lepidoptera* found at high altitude [30], has been used in patients with chronic kidney disease and animal model of kidney ischemia/reperfusion [31]. It has been documented that *Cordyceps sinesi* had strong anti-oxidative activity and could improve renal function after kidney ischemia/reperfusion injury. Moreover this function might be related to its regulation of inflammatory cytokines such as chemoatractant protein-1 and tumor necrosis factor-α [32]. Other Chinese herbs such as *Rheum palmatum* (chishao) [33], *Salviae miltiorrhizae* (danshen) [34], *Astragalus membranaceus* (huangqi) and *Angelica sinensis* (dangui) [35] could also have anti-oxidative activity and help to recover kidney function in ischemia/reperfusion injury or nephrotic syndrome. Moreover, a traditional Chinese herbal formula - Sairei-to (chailingtang) has been shown to have protective effects on kidney injury due to its suppression of mesangial cell proliferation [36]. Therefore, not only single herb but also combination of herbs has renal protective property. Moreover, a Korean herbal combination (KIOM-79) was able to reduce advance glycation end product accumulation in diabetic rats [37]. This finding indicates that herbal products might also protect kidney injury in patients with diabetes. The current finding in our study also strongly supports that herbal product might reduce podocalyxin expression to prevent diabetic induced kidney injury.

## Conclusions

Podocalyxin expression was significantly reduced in the kidney of STZ induced diabetes. A unique formulation of Chinese herbs (Yishen capsule) used in our hospital could restore the expression of podocalyxin in the kidney induced by STZ and improve microalbuminuria in the rats. However, this formulation was not able to reduce blood glucose level in STZ induced rats.

## Competing interests

The author(s) declare that they have no competing interests.

## Authors’ contributions

JF designed the study and coordinated the experiments and draft of the manuscript. HW carried out the animal study. YS participated in the experimental design and data analyses. XZ involved in the immunohistochemical staining. WL conducted the extraction of RNA and RT-PCR. QC participated in electron microscopic experiment. RW participated in the design and analysis of the experiment. YG involved in discussion of experiment and draft of the manuscript. All authors read and approved the final manuscript.

## Pre-publication history

The pre-publication history for this paper can be accessed here:

http://www.biomedcentral.com/1472-6882/13/76/prepub
